# A Novel Reassortant Avian H7N6 Influenza Virus Is Transmissible in Guinea Pigs via Respiratory Droplets

**DOI:** 10.3389/fmicb.2019.00018

**Published:** 2019-01-22

**Authors:** Zongzheng Zhao, Lina Liu, Zhendong Guo, Chunmao Zhang, Zhongyi Wang, Guoyuan Wen, Wenting Zhang, Yu Shang, Tengfei Zhang, Zuwu Jiao, Ligong Chen, Cheng Zhang, Huan Cui, Meilin Jin, Chengyu Wang, Qingping Luo, Huabin Shao

**Affiliations:** ^1^Institute of Animal Husbandry and Veterinary Sciences, Hubei Academy of Agricultural Sciences, Wuhan, China; ^2^Institute of Military Veterinary, Academy of Military Medical Sciences, Changchun, China; ^3^College of Veterinary Medicine, Hebei Agricultural University, Baoding, China; ^4^College of Veterinary Medicine, Huazhong Agricultural University, Wuhan, China

**Keywords:** avian H7N6 influenza A virus, reassortment, receptor binding, pathogenicity, transmissibility

## Abstract

Since 2013, H7N9 and H5N6 avian influenza viruses (AIVs) have caused sporadic human infections and deaths and continued to circulate in the poultry industry. Since 2014, H7N6 viruses which might be reassortants of H7N9 and H5N6 viruses, have been isolated in China. However, the biological properties of H7N6 viruses are unknown. Here, we characterize the receptor binding preference, pathogenicity and transmissibility of a H7N6 virus A/chicken/Hubei/00095/2017(H7N6) (abbreviated HB95), and a closely related H7N9 virus, A/chicken/Hubei/00093/2017(H7N9) (abbreviated HB93), which were isolated from poultry in Hubei Province, China, in 2017. Phylogenetic analyses demonstrated that the hemagglutinin (HA) gene of HB95 is closely related to those of HB93 and human-origin H7N9 viruses, and that the neuraminidase (NA) gene of HB95 shared the highest nucleotide similarity with those of H5N6 viruses. HB95 and HB93 had binding affinity for human-like α2, 6-linked sialic acid receptors and were virulent in mice without prior adaptation. In addition, in guinea pig model, HB93 was transmissible by direct contact, but HB95 was transmissible via respiratory droplets. These results revealed the potential threat to public health posed by H7N6 influenza viruses and emphasized the need for continued surveillance of the circulation of this subtype in poultry.

## Introduction

Influenza A viruses are negative-sense RNA viruses of the family Orthomyxoviridae. Based on the antigenic differences in their surface glycoproteins, hemagglutinin (HA) and neuraminidase (NA), influenza A viruses are currently categorized into 18 different HA and 11 different NA subtypes (Tong et al., 2013), but only viruses with HA subtypes H1, H2, and H3 and NA subtypes N1 and N2 are known to cause influenza pandemics in humans (Kilbourne, 2006; Kalthoff et al., 2010; Li and Chen, 2014). Avian influenza viruses (AIVs) can be classified into low and highly pathogenic viruses according to the monobasic or polybasic nature of the basic amino acids in the HA cleavage site and the mortality rate in poultry. Low pathogenic avian influenza viruses (LPAIVs) lead to restricted infection and mild disease in poultry, while highly pathogenic avian influenza viruses (HPAIVs) lead to systemic infection and a mortality rate of nearly 100% in poultry. So far, only viruses with subtypes H5 and H7 are called HPAIVs (Kalthoff et al., 2010), but these subtypes also include some LPAIVs.

AIVs, including H5N1, H5N6, H7N2, H7N3, H7N7, H7N9, H9N2, and H10N8 virus subtypes (Bender et al., 1999; Lin et al., 2000; Fouchier et al., 2004; Centers for Disease Control et al., 2012; Ostrowsky et al., 2012; Gao et al., 2013; Chen et al., 2014; Mok et al., 2015; Yang et al., 2015), have occasionally broken the species barrier and infected humans but have not been able to disseminate. The major reason underlying their limited human transmissibility was the weak affinity of these viruses for human-like receptors (Imai and Kawaoka, 2012). However, Human-type receptor recognition by AIVs is probably necessary but not sufficient for their transmission via respiratory droplet in a ferret model. An H5N1 virus that recognizes both receptors is not transmissible via respiratory droplets between ferrets (Maines et al., 2006). Therefore, other important phenotypes, such as HA stability and replicative ability linked to high polymerase activity, also have been shown to be needed for efficient airborne transmissibility in the ferret model (Imai and Kawaoka, 2012; Linster et al., 2014). Poultry function as “vessels” for the transmission of many AIV subtypes from poultry to humans. Since 2013, H7N9 and H5N6 subtypes of AIVs have been reported to spread to humans in China (Hu et al., 2014; Yang et al., 2015; Zhang et al., 2016).

H7 subtypes of AIVs have circulated in poultry in China since 2002 (Li et al., 2006), and human infections with H7N9 subtype viruses were reported in China in 2013 (Gao et al., 2013). In recent years, H7N9 has shown an increasing trend in prevalence among AIVs in domestic poultry in China (Shi et al., 2017). Since 2000, N6 subtypes of AIVs have been found in poultry in China. N6 subtype influenza viruses circulated widely in duck populations in southern and eastern China in 2006 (Bi et al., 2016), and the first human infection with an N6 (H5N6) subtype virus was reported in 2014 (Yang et al., 2015; Zhang et al., 2016; He et al., 2018). Notably, H5N6 viruses have become a dominant subtype in poultry in southern China (Bi et al., 2016). These reports suggest that H7N9 and H5N6 subtypes of AIVs can to cross the species barrier and infect humans, emphasizing the need for continued surveillance of the circulation of these AIV subtypes in poultry.

In China, H7N6 subtype influenza viruses were first isolated in 2007. The first isolate was found in Yunnan Province in 2007 (Lam et al., 2013). The second isolates, which might be reassortants of H7N9 and H5N6 viruses, were found in Jiangxi Province in 2014 (Lam et al., 2015). The third isolates were found in Zhejiang Province in 2016 (Wu et al., 2017). In this study, the zoonotic capability and pathogenicity of a reassortant H7N6 virus (HB95), which have acquired its genes from H7N9 and H5N6 viruses, is characterized and evaluated as a potential threat to human health.

## Materials and Methods

### Ethics Statement

All animal studies were conducted in strict accordance with the animal welfare guidelines of the World Organization for Animal Health. The animal studies were performed according to the protocols approved by the Hubei Provincial Animal Care and Use Committee (approval number SCXK 2015-0018). All experiments with the viruses were performed in biosecurity level 3 laboratory approved by Huazhong Agricultural University.

### Viruses

The viruses HB95, HB93 and A/chicken/Hubei/00019/2017(H5N6) (abbreviated CK19) used in this study were isolated from chickens during surveillance for AIVs in China in 2017. A/Mexico/4486/2009(H1N1) (abbreviated Mex) was generated by an eight plasmid reverse genetics system that. The viruses were grown in 9-day-old specific pathogen free eggs for 72 h at 37°C (Merial Vital Laboratory Animal Technology Company, Beijing, China) and stored at –80°C.

### Phylogenetic and Sequence Analysis

Viral RNA was extracted from allantoic fluid using TRIzol reagent (Invitrogen) and reverse transcribed into cDNAs using the primer Uni12 (5′-AGC RAA AGC AGG-3′). The PCR products of eight fragments of the viruses were amplified by PCR using specific virus primers as described by Hoffmann et al. (2001). The PCR products were purified and sequenced by Comate Bioscience Company Limited. All the sequence data were analyzed with the SEQMAN program (DNASTAR, Madison, WI, United States). All reference sequences used in this study were obtained from the National Center for Biotechnology Information (NCBI) GenBank database. Phylogenic analysis was performed by the distance based neighbor-joining method using MEGA7.0.21 software.

### Receptor Binding Specificity Assay

The receptor-binding specificities of the three viruses were determined by HA assays with 1% chicken red blood cells (cRBCs). For the HA assay, sialic acid residues were enzymatically removed from cRBCs by incubating the cells with 50 mU of *Vibrio cholerae* neuraminidase (VCNA, Roche, San Francisco, CA, United States) at 37°C for 1 h, followed by resialylation using either α2-,6-N-sialyltransferase or α2-,3-N-sialyltransferase (Sigma-Aldrich, St. Louis, MO, United States) at 37°C for 4 h. The sample was then washed two times with phosphate-buffered saline (PBS), centrifuged at 1500 rpm for 5 min each time, adjusted to a final working concentration (1%) with PBS, and stored at 4°C. For the HA assay, viruses were serially diluted 2-fold with 50 μL of PBS and mixed with 50 μL of a 1% RBC suspension in a 96-well plate. HA titers were read after 1 h at 4°C temperature.

### Cell Culture and Growth Curves

Madin-Darby canine kidney (MDCK) cells were obtained from the American Type Culture Collection (ATCC) and maintained in Dulbecco’s modified Eagle’s medium (DMEM; Invitrogen, Carlsbad, CA, United States) with supplemented 10% fetal bovine serum (FBS; Gibco, Auckland, New Zealand). The growth kinetics of HB95 and HB93 were determined by inoculating MDCK cells at a multiplicity of infection (MOI) of 0.001 50% tissue culture infectious dose (TCID_50_) per cell. One hour after inoculation, the cells were washed twice with PBS, and fresh medium supplemented with 1 μg/mL tosyl phenylalanyl chloromethyl ketone (TPCK) and trypsin (Sigma-Aldrich, St. Louis, MO, United States) was added. The supernatants were sampled at 12, 24, 36, and 48 h post-infection (hpi). The virus titers were determined by calculating the log_10_ TCID_50_/mL in MDCK cells. The TCID_50_ values were calculated according to the method of Reed and Muench.

### Mouse Experiments

Groups of five six-week-old female BALB/c mice (Merial Vital Laboratory Animal Technology Company, Beijing, China) were anesthetized with ether and intranasally inoculated with 50 μL of a 10^5.0^ EID_50_ solution of HB95 or HB93. The weight loss and mortality of mice in these groups were monitored daily for 14 days. Mice that lost > 30% of their original body weight were humanely euthanized.

To assess the growth characteristics of the three viruses and the pathological changes in the lungs of the infected mice, two groups of 12 mice were anesthetized with ether and intranasally instilled with 10^5.0^ EID_50_ of either HB95 or HB93, while another three mice intranasally instilled with PBS were used as controls. Three mice were euthanized at 3 and 5 days post-infection (dpi). The lungs of the infected mice were removed to determine the virus titers. Briefly, the lung tissues were weighed, and 0.1 g of each tissue was placed into 1 mL of PBS containing 100 U/mL penicillin, generating 10% weight/volume lung homogenates. The tissue samples were homogenized by Tissue Lyser (QIAGEN, Germany) and centrifuged at 12,000 rpm. Then the supernatants were collected and inoculated into 9-day-old embryonated eggs. After 72 h incubation at 37°C, HA activity was tested and the EID_50_ was determined by the Reed and Muench method. The lungs of the infected mice euthanized at 5 dpi were fixed in formalin, and the fixed tissues were embedded in paraffin and stained with hematoxylin and eosin for pathological examination.

### Guinea Pig Experiments

Hartley strain female guinea pigs weighing 300 to 350 g (Merial Vital Laboratory Animal Technology Company, Beijing, China), confirmed to be seronegative to influenza viruses prior to the experiment, were used in these studies. In the transmission studies, groups of three guinea pigs were anesthetized with ether and intranasally inoculated with 300 μL of a 10^5.0^ EID_50_ solution of the test virus and housed in a cage placed in an isolator. The next day, three naive guinea pigs were individually paired and cohoused with an infected guinea pig for the direct contact transmission studies, and another naive guinea pig was housed in a wire frame cage adjacent to the infected guinea pig for the aerosol transmission studies. The distance between the cages of the infected and aerosol-contact guinea pigs was 5-cm. To monitor virus shedding, nasal washes were collected form all animals at 2, 4, 6, and 8 dpi and titrated.

### Statistics Analysis

Statistically significant differences were determined using one-way analysis of variance (ANOVA) with GraphPad Prism software (San Diego, CA, United States). All assays were run in triplicate, and the data are representative of at least 3 separate experiments. The error bars indicate the standard deviation.

## Results

### The H7N6 Virus Likely Arose From Reassortment Between H7N9 and H5N6 Viruses

**Figure 1:**
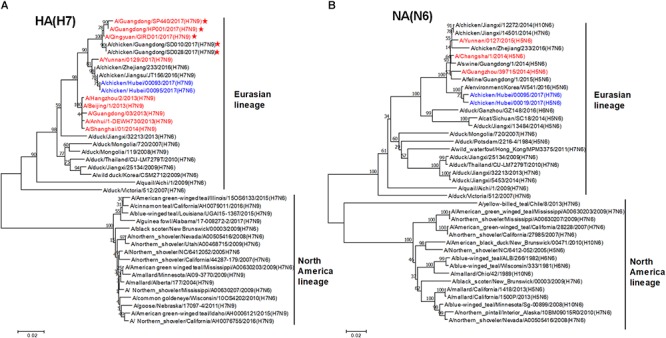
Phylogenetic analysis of HA and NA genes. Phylogenetic trees of the HA **(A)** and NA **(B)** genes were constructed using the distance-based neighbor joining method in MEGA7.0.21 software. Sequences of viruses with names were downloaded from NCBI. The reliability of the trees was assessed by bootstrap analysis. Horizontal distances are proportional to genetic distances. The viruses analyzed in the present study is indicated in blue. Human viruses are indicated in red. Viruses labeled with a red star contain the four-amino acid (-KRTA-) insertion in their HA genes.

To trace the origin of HB95, all gene segments were sequenced and compared with nucleotide sequences available in GenBank databases. Phylogenetic analysis demonstrated that the HA genes of HB95 belonged to the Eurasian lineage and were closely related to those of HB93 and human isolate H7N9 viruses (Figure 1A). The NA genes of HB95 were derived from N6-like Eurasian virus lineages and were similar to those of the human isolates A/Changsha/1/2014 (H5N6), A/Guangzhou/39715/2014 (H5N6) and A/Yunnan/0127/2015 (H5N6) (Figure 1B).

Sequence analysis showed that seven genes of HB95 shared homology with those of HB93, excepted for the NA genes. The HA, PB2, PB1, PA, and NP genes of HB95 shared greater than 99% nucleotide similarity with those of HB93, and the M and NS genes of HB95 shared 97 and 94% nucleotide similarity, respectively, with those of HB93. The HA proteins of HB95 and HB93 showed the LPAIVs amino acid sequence PEIPK↓GRGLF at HA1 and HA2 cleavage site. The HB95 NA gene shared greater than 99% nucleotide similarity with those of CK19 and A/environment/Korea/W541/2016(H5N6), and 95–97% nucleotide identity with those of the human isolates A/Changsha/1/2014 (H5N6), A/Guangzhou/39715/2014 (H5N6) and A/Yunnan/0127/2015 (H5N6). HB95 exhibited an 11-amino acid deletion in the NA stalk regions (positions 59 to 69), which was similar to that in the human isolate H5N6 viruses. Based on these findings, the HB95 virus in this study might have arisen from H7N9 and H5N6.

### The H7N6 Virus Exhibited Comparable Binding Affinity for Avian and Human Sialic Acid Receptors

**Figure 2:**
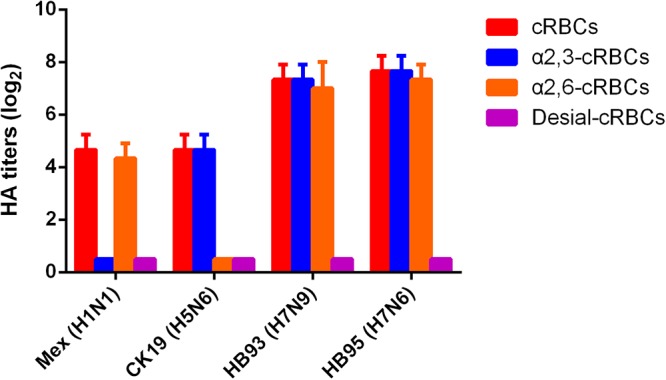
Agglutination activities of the H7N6 virus with various red blood cells. Four types of chicken red blood cells (cRBCs): a, cRBCs. b, α-2,6 cRBCs (treated with VCNA and resialylated with α-2,6 glycans). c, α-2,3 cRBCs (treated with VCNA and resialylated with α-2,3 glycans), d, desialylated (Desial) cRBCs (treated with VCNA). The HA titers showed the agglutination activities of HB95, HB93, CK19 and Mex for the four types of cRBCs. The reported values are presented as the means and standard deviations of three independent experiments.

The receptor binding specificity of influenza A viruses is a critical determinant of cross-species transmission (Connor et al., 1994; Matrosovich et al., 2000). We thus evaluated the receptor binding specificity of the HB95, HB93, and CK19 viruses. The HA titers are shown in Figure 2 and are representative of the results of three separate experiments. The surface of cRBCs contains avian-like (α2, 3-linked sialic acid, SA α2,3Gal) and human-like (α2, 6-linked sialic acid, SA α2,6Gal) receptors, the surface of cRBCs treated with VCNA contains no receptors (Desialylation-cRBCs), and the surface of resialylated cRBCs contains either SAα2,6Gal (α2,6-cRBCs) or SAα2,3Gal (α2,3-cRBCs). The HB95 and HB93 viruses bound to both α2,6-resialylated and α2,3-resialylated cRBCs at comparable levels (Figure 2), while Mex or CK19 bound only to α2,6-resialylated cRBCs or α2,3-resialylated cRBCs. These results suggested that HB95 and HB93 were able to bind to human-like receptors.

### The H7N6 Virus Showed Higher Pathogenicity Than the H7N9 Virus in Mice

**Figure 3:**
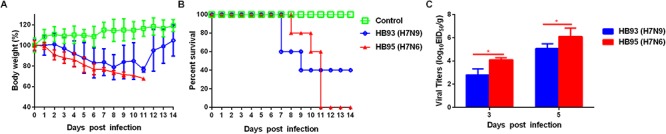
Pathogenicity in mice. Five mice per group were intranasally inoculated with 10^5.0^ EID_50_ of HB95 or HB93. **(A)** Mouse body weights were monitored daily for 14 days. The values are the average scores of the overall body weight loss with respect to the initial body weights, ± standard deviations (SDs). **(B)** The survival percentages were calculated by observing the infected mice. **(C)** Lungs were collected from mice inoculated with 10^5.0^ EID_50_ of HB95 or HB93 at 3 and 5 dpi (*n* = 3), and virus titers were determined in 9-day-old specific pathogen free embryonated eggs (^∗^*P* < 0.05).

To evaluate the pathogenicity of HB95 and HB93 viruses in mammalian hosts, five six-week-old female BALB/c mice were anesthetized with ether and intranasally instilled with 50 μL of a 10^5.0^ EID_50_ solution of HB95 or HB93. Mice infected with HB95 and mice infected with HB93 showed a weight loss of approximately 30 and 20% and displayed a mortality rate of 100 or 60%, respectively (Figures 3A,B).

We next compared the virus titers of HB95 and HB93 in the lungs of the infected mice. Both viruses were readily detected in the lungs of mice, with titers ranging from 10^2.8^ to 10^6.3^ EID_50_/g (Figure 3C). In HB95-infected mice, the virus titers were 10^4.1^EID_50_/g at 3 dpi and 10^6.3^ EID_50_/g at 5 dpi, approximately 10-fold higher than the 10^2.8^ EID_50_/g at 3 dpi and 10^5.1^ EID_50_/g at 5 dpi exhibited by HB93 (*P* < 0.05, *n* = 3).

**Figure 4:**
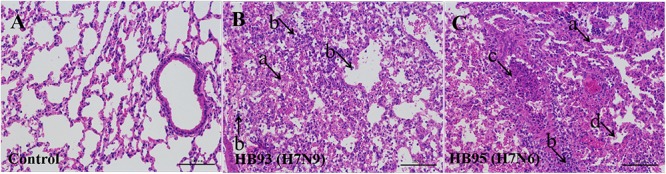
Histopathological analysis. At 5 dpi, lungs were collected from mice inoculated with 10^5.0^ EID_50_ of HB95 or HB93, and were fixed with formalin, embedded in paraffin and stained with hematoxylin and eosin. The images were obtained at a magnification of × 20. **(A–C)** show the pathological changes in the lungs of mice inoculated with PBS, HB95 or HB93, respectively. (arrow a) alveolar wall thickening, (arrow b) infiltration of inflammatory cells in the alveolar space, (arrow c) infiltration of inflammatory cells in the bronchus, (arrow d) pulmonary edema. The scale bar represents 100 μm.

Additionally, we performed histopathological analysis. The lungs of the negative control mice exhibited large air spaces and thin alveolar walls, indicating of a normal phenotype (Figure 4A), but the infected mice showed alveolar wall thickening, inflammatory cell and pulmonary edema at 5 dpi (Figures 4B,C). Thus, histological analysis revealed pathological changes in both HB95- and HB93-infected mice.

In summary, based on the results of the mouse studies, the HB95 virus showed higher pathogenicity than the HB93 virus in mice.

### H7N6 Displayed Advantageous Growth Properties in MDCK Cells

**Figure 5:**
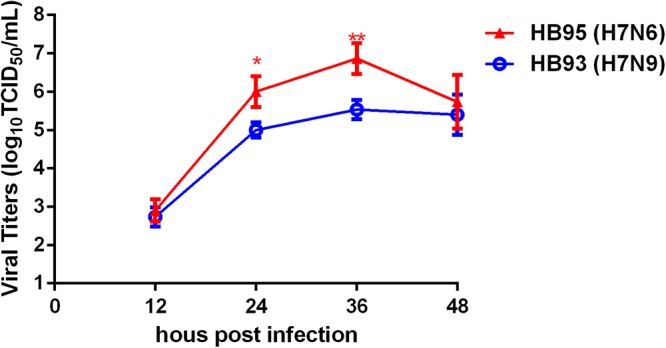
Characterization grow kinetics in MDCK cells. Growth kinetics of HB95 and HB93. MDCK cells were infected with HB95 or HB93 at an MOI of 0.001 TCID_50_ per cell and treated with 1 μg/mL TPCK. At the indicated hpi, the virus titers in the supernatants were determined with the MDCK cells. The reported values are the means and standard deviations of three independent experiments (^∗^*P* < 0.05, ^∗∗^*P* < 0.01).

To investigate the growth kinetics of HB95 and HB93, we compared the multicycle growth in MDCK cells. The virus titers of HB95 peaked at 10^6.8^ TCID_50_/mL and those of HB93 peaked at 10^5.5^ TCID_50_/mL at 36 hpi (Figure 5). HB95 exhibited a significantly increased growth ability, with virus titers more than 10-fold higher than those of HB93 (^∗^*P* < 0.5, ^∗∗^*P* < 0.01, *n* = 3). These results indicated that compared with HB93, HB95 exhibited advantageous growth properties in MDCK cells.

### H7N6 Is Transmissible in Guinea Pigs via Respiratory Droplets

**Figure 6:**
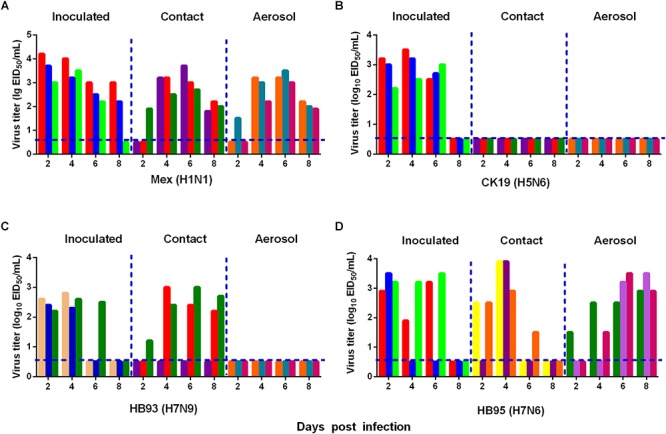
Horizontal transmissions of the viruses in guinea pigs. Groups of three guinea pigs seronegative for influenza viruses were inoculated with 10^5.0^ EID_50_ of the test viruses. The next day, the three inoculated guinea pigs were individually paired by cohousing with a direct-contact guinea pig; in addition, an aerosol contact guinea pig was housed in a wire frame cage adjacent to that of the infected guinea pig. The distance between the cages of the infected and aerosol-contact guinea pigs was 5 cm apart. Nasal washes were collected from all animals for virus shedding detection every other day beginning on day 2 after the initial infection. Each color bar represents the virus titer in an individual animal. The dashed lines indicate the lower limit of virus detection. Horizontal transmissions of the Mex **(A)**, CK19 **(B)**, HB93 **(C)**, and HB95 **(D)** viruses in guinea pigs.

We also measured the transmissibility of HB95 and HB93 in guinea pigs as we previously reported (Zhang et al., 2017; Zhao et al., 2017). Groups of three guinea pigs were intranasally inoculated with a 10^5.0^ EID_50_ solution of the test virus. Twenty-four hours later, three inoculated guinea pigs were individually paired and cohoused with a direct contact guinea pig; in addition, an aerosol contact guinea pig was housed in a wire frame cage adjacent to that of the infected guinea pig. The distance between cages of the infected and aerosol contact guinea pigs was 5 cm apart. To monitor virus shedding, nasal washes were collected from all inoculated, direct contact and aerosol transmission groups and titrated for virus in 9-day-old embryonated eggs. In CK19 groups, viruses were detected only in nasal washes from the inoculated group, indicating no transmission (three animals were examined for each route) (Figure 6B). HB93 was transmitted to two guinea pigs via the direct contact route but not via the aerosol route (three animals were examined for each route) (Figure 6C). However, either HB95 or Mex was transmissible via the direct contact and aerosol-treated route (three animals were examined for each route) (Figures 6A,D), indicating that the HB95 and Mex viruses transmit efficiently via respiratory droplets. The recipient guinea pigs also showed clinical signs and symptoms such as depression, shivering and shortness of breath, but neither HB95 nor HB93 resulted in lethal infections. The guinea pigs that did not shed virus following exposure (either direct or aerosol transmission contacts) remained seronegative at the end of the study. These findings demonstrated that HB95 was transmissible among guinea pigs via respiratory droplets.

## Discussion

Influenza A viruses are constantly changing. The exchange of intact gene segments through reassortment plays a major role in generating novel epidemic, pandemic, and zoonotic influenza viral strains (Lowen, 2017; Richard et al., 2017). In the present study, we focused on the biological properties of a H7N6 virus. Phylogenetic and sequence analyses showed that HB95 might receive its genes from HB93, except for the NA gene; thus, the pathogenicity and transmissibility of these two viruses were studied.

AIVs that cross the species barrier to infect mammals usually feature the evolution of the receptor binding preference from avian-like receptors to both avian and human-like receptors (Suzuki et al., 2000; Ha et al., 2001; Parrish and Kawaoka, 2005). The HA gene of the influenza virus contains receptor binding sites and determines the receptor binding specificity. The molecular markers of Gln(Q) or Leu(L) at HA residue 226 (H3 numbering) are avian and human specific residues, respectively. The combination of Ser (S) or Gly (G) at residue 228 with Q or L at residue 226 can switch the binding preference from avian receptors to human receptors, with two changes (Naeve et al., 1984; Connor et al., 1994). Combinations of HA amino acid 226Q/228G, 226L/ 228S or 226L/ 228G are commonly observed in avian, human, or swine influenza viruses, respectively. Viruses isolated from avian, human and swine sources show a correlation between receptor binding preference and species of origin: avian isolates (226Q/228G) prefer α-2,3-linked sialic acid receptors, human isolates (226L/ 228S) prefer α-2,6 -linked sialic acid receptors, and swine isolates (226L/ 228G) prefer both receptors (Rogers and D’Souza, 1989; Connor et al., 1994; Ito et al., 1998; Matrosovich et al., 1999). The HA 158D increases the receptor binding affinity of influenza viruses for α2,6-linked sialic acid receptors without reducing their binding affinity for α2, 3-linked sialic acid receptors (Stevens et al., 2008; Gao et al., 2009). Residues 190E and 225G in the HA receptor-binding site are highly conserved among avian viruses and appear important for the affinity of these viruses for α2,3-linked sialic acid receptors (Matrosovich et al., 2000). In this study, amino acids 158D/226L in the HA of HB95 and HB93 might contribute their binding to both avian-like and human-like receptors. However, amino acids 158N/226Q in the HA of CK19 might lead CK19 to recognize only avian-like receptors.

H5N1 hybrid viruses bearing 2009/H1N1 virus genes were reported to be transmitted by respiratory droplets in a guinea pig model (Zhang et al., 2013). In addition, H5N6 and H7N9 viruses were transmissible by direct contact in a guinea pig model (Gabbard et al., 2014; Zhao et al., 2017). In our mouse challenge study, HB95 exhibited a higher pathogenicity than the closely related HB93 (Figure 3). In the transmission study, HB93, similar to other previously reported H7N9 viruses, was transmissible by direct contact in the guinea pig model (Gabbard et al., 2014). Compared to the closely related HB93, HB95 displayed an advantage for transmission via respiratory droplets in the guinea pig model (Figure 6). Several research groups have evaluated the transmissibility of H7N9 viruses in guinea pig model and some H7N9 viruses strains indeed transmit via aerosol in guinea pigs (Kong et al., 2015; Yang et al., 2018). But the HB93 virus in our study only transmitted via direct contact but not via respiratory droplets in guinea pigs. We do not yet know the full range of factors that limited the airborne transmission of HB93, but several important phenotypes might contribute its limited transmissibility in the guinea pig model. Such as low viral shedding in guinea pigs, the avian H7N9 A/chicken/Hunan/S1220/2017(H7N9) virus in Yang et al. study which was airborne transmissible peaks more than 10^6^ EID_50_/mL in guinea pigs, with virus titers more than 1000-fold higher than 10^3^ EID_50_/mL of HB93 in our study; amino acid changes in H7N9 viruses which would make the influenza viruses transmissible in animal model, such as amino acids in PB2, lysine at position 627K and 701N which some human H7N9 isolates encode, are important for influenza transmission in ferret and guinea pig models (Watanabe et al., 2014), the H7N9 (HB93) virus in our study contains neither 627K nor 701N in the PB2 gene which might lead the limited transmissibility in the guinea pig model. The Ferrets have been widely used as animal models for H7N9 influenza virus transmission studies and some H7N9 viruses exhibit limited transmission via respiratory droplets (Richard et al., 2013), but airborne transmissible in the guinea pig model (Kong et al., 2015; Yang et al., 2018). The HB93 virus in this study only exhibited direct contact transmission but no airborne transmission in guinea pig model; the HB95 also contains neither 627K nor 701N in the PB2 gene, but exhibited airborne transmission in guinea pig model, thus we are going to study on the molecule mechanism why HB93 is not airborne transmissible in the guinea pig model. HB95 also exhibited higher growth kinetics in MDCK cells and mice and guinea pigs than HB93 (Figures 3,5,6); thus, the increased growth kinetics may contribute to the greater pathogenicity in mice and transmissibility in guinea pigs, but may not be the sole underlying mechanism. Importantly, some H7N9 viruses obtained an insertion of four amino acids in their HA) cleavage site and became highly lethal in mice (Imai et al., 2017; Shi et al., 2017). Thus, attention should also be devoted to further mutations in the HA cleavage site of H7N6 viruses, which may cause the virus to become highly pathogenic.

In summary, a reassortant H7N6 virus was isolated; this virus exhibited comparable binding affinity for both avian-like and human-like receptors, and displayed efficient airborne transmission in the guinea pig model. Chickens may have played an important role in the generation of the novel reassortant H7N6 virus. Our study provided insight into H7N6 viruses in chickens in China. Notably, low pathogenic H7N6 viruses can mutate to high pathogenicity subtypes during circulation in domestic chicken, thus posing an increased threat to human health. Therefore, constant surveillance of H7N6 viruses highly recommended to prevent a possible pandemic.

## Author Contributions

ZZ and LL designed and supervised the study and wrote the grant application. ZG, ChuZ, ZW, GW, WZ, YS, TZ, and ZJ performed the receptor binding specificity assay, cell culture experiments, and animal studies. LC, CheZ, and HC performed the histopathological analyses. MJ, CW, QL, and HS designed the work and revised it critically. All authors had read manuscript and approved its final version.

## Conflict of Interest Statement

The authors declare that the research was conducted in the absence of any commercial or financial relationships that could be construed as a potential conflict of interest.

## References

[B1] Bender C. Hall H. Huang J. Klimov A. Cox N. Hay A. (1999). Characterization of the surface proteins of influenza A (H5N1) viruses isolated from humans in 1997-1998. *Virology* 254 115–123. 10.1006/viro.1998.9529 9927579

[B2] Bi Y. Chen Q. Wang Q. Chen J. Jin T. Wong G. (2016). Genesis, evolution and prevalence of H5N6 avian influenza viruses in China. *Cell Host Microbe* 20 810–821. 10.1016/j.chom.2016.10.022 27916476

[B3] Centers for Disease Control and Prevention [CDC] (2012). Notes from the field: highly pathogenic avian influenza A (H7N3) virus infection in two poultry workers–Jalisco, Mexico, July 2012. *MMWR Morb. Mortal. Wkly. Rep.* 61 726–727. 22971746

[B4] Chen H. Yuan H. Gao R. Zhang J. Wang D. Xiong Y. (2014). Clinical and epidemiological characteristics of a fatal case of avian influenza A H10N8 virus infection: a descriptive study. *Lancet* 383 714–721. 10.1016/S0140-6736(14)60111-2 24507376

[B5] Connor R. J. Kawaoka Y. Webster R. G. Paulson J. C. (1994). Receptor specificity in human, avian, and equine H2 and H3 influenza virus isolates. *Virology* 205 17–23. 10.1006/viro.1994.1615 7975212

[B6] Fouchier R. A. Schneeberger P. M. Rozendaal F. W. Broekman J. M. Kemink S. A. Munster V. (2004). Avian influenza A virus (H7N7) associated with human conjunctivitis and a fatal case of acute respiratory distress syndrome. *Proc. Natl. Acad. Sci. U.S.A.* 101 1356–1361. 10.1073/pnas.0308352100 14745020 PMC337057

[B7] Gabbard J. D. Dlugolenski D. Van Riel D. Marshall N. Galloway S. E. Howerth E. W. (2014). Novel H7N9 influenza virus shows low infectious dose, high growth rate, and efficient contact transmission in the guinea pig model. *J. Virol.* 88 1502–1512. 10.1128/JVI.02959-13 24227867 PMC3911619

[B8] Gao R. Cao B. Hu Y. Feng Z. Wang D. Hu W. (2013). Human infection with a novel avian-origin influenza A (H7N9) virus. *N. Engl. J. Med.* 368 1888–1897. 10.1056/NEJMoa1304459 23577628

[B9] Gao Y. Zhang Y. Shinya K. Deng G. Jiang Y. Li Z. (2009). Identification of amino acids in HA and PB2 critical for the transmission of H5N1 avian influenza viruses in a mammalian host. *PLoS Pathog* 5:e1000709. 10.1371/journal.ppat.1000709 20041223 PMC2791199

[B10] Ha Y. Stevens D. J. Skehel J. J. Wiley D. C. (2001). X-ray structures of H5 avian and H9 swine influenza virus hemagglutinins bound to avian and human receptor analogs. *Proc. Natl. Acad. Sci. U.S.A.* 98 11181–11186. 10.1073/pnas.201401198 11562490 PMC58807

[B11] He J. Liu B. Y. Gong L. Chen Z. Chen X. L. Hou S. (2018). Genetic characterization of the first detected human case of avian influenza A (H5N6) in Anhui Province, East China. *Sci. Rep.* 8:15282. 10.1038/s41598-018-33356-4 30327485 PMC6191424

[B12] Hoffmann E. Stech J. Guan Y. Webster R. G. Perez D. R. (2001). Universal primer set for the full-length amplification of all influenza A viruses. *Arch. Virol.* 146 2275–2289. 10.1007/s007050170002 11811679

[B13] Hu J. Zhu Y. Zhao B. Li J. Liu L. Gu K. (2014). Limited human-to-human transmission of avian influenza A(H7N9) virus, Shanghai, China, March to April 2013. *Euro Surveill.* 19:20838. 10.2807/1560-7917.ES2014.19.25.20838 24993556

[B14] Imai M. Kawaoka Y. (2012). The role of receptor binding specificity in interspecies transmission of influenza viruses. *Curr. Opin. Virol.* 2 160–167. 10.1016/j.coviro.2012.03.003 22445963 PMC5605752

[B15] Imai M. Watanabe T. Kiso M. Nakajima N. Yamayoshi S. Iwatsuki-Horimoto K. (2017). A highly pathogenic avian H7N9 influenza virus isolated from a human is lethal in some ferrets infected via respiratory droplets. *Cell Host Microbe* 22 615.e8–626.e8. 10.1016/j.chom.2017.09.008 29056430 PMC5721358

[B16] Ito T. Couceiro J. N. Kelm S. Baum L. G. Krauss S. Castrucci M. R. (1998). Molecular basis for the generation in pigs of influenza A viruses with pandemic potential. *J. Virol.* 72 7367–7373. 9696833 10.1128/jvi.72.9.7367-7373.1998 PMC109961

[B17] Kalthoff D. Globig A. Beer M. (2010). (Highly pathogenic) avian influenza as a zoonotic agent. *Vet. Microbiol.* 140 237–245. 10.1016/j.vetmic.2009.08.022 19782482

[B18] Kilbourne E. D. (2006). Influenza pandemics of the 20th century. *Emerg. Infect. Dis.* 12 9–14. 10.3201/eid1201.051254 16494710 PMC3291411

[B19] Kong H. Zhang Q. Gu C. Shi J. Deng G. Ma S. (2015). A live attenuated vaccine prevents replication and transmission of H7N9 virus in mammals. *Sci. Rep.* 5:11233. 10.1038/srep11233 26058711 PMC4462025

[B20] Lam T. T. Wang J. Shen Y. Zhou B. Duan L. Cheung C. L. (2013). The genesis and source of the H7N9 influenza viruses causing human infections in China. *Nature* 502 241–244. 10.1038/nature12515 23965623 PMC3801098

[B21] Lam T. T. Zhou B. Wang J. Chai Y. Shen Y. Chen X. (2015). Dissemination, divergence and establishment of H7N9 influenza viruses in China. *Nature* 522 102–105. 10.1038/nature14348 25762140

[B22] Li C. Chen H. (2014). Enhancement of influenza virus transmission by gene reassortment. *Curr. Top. Microbiol. Immunol.* 385 185–204. 10.1007/82-2014-389 25048543

[B23] Li Y. Li C. Liu L. Wang H. Wang C. Tian G. (2006). Characterization of an avian influenza virus of subtype H7N2 isolated from chickens in northern China. *Virus Genes* 33 117–122. 10.1007/s11262-005-0042-8 16791426

[B24] Lin Y. P. Shaw M. Gregory V. Cameron K. Lim W. Klimov A. (2000). Avian-to-human transmission of H9N2 subtype influenza A viruses: relationship between H9N2 and H5N1 human isolates. *Proc. Natl. Acad. Sci. U.S.A.* 97 9654–9658. 10.1073/pnas.160270697 10920197 PMC16920

[B25] Linster M. van Boheemen S. de Graaf M. Schrauwen E. J. A. Lexmond P. Mänz B. (2014). Identification, characterization, and natural selection of mutations driving airborne transmission of A/H5N1 virus. *Cell* 157 329–339. 10.1016/j.cell.2014.02.040 24725402 PMC4003409

[B26] Lowen C. (2017). Constraints, drivers, and implications of influenza A virus reassortment. *Annu. Rev. Virol.* 4 105–121. 10.1146/annurev-virology-101416-041726 28548881

[B27] Maines T. R. Chen L. M. Matsuoka Y. Chen H. Rowe T. Ortin J. (2006). Lack of transmission of H5N1 avian-human reassortant influenza viruses in a ferret model. *Proc. Natl. Acad. Sci. U.S.A.* 103 12121–12126. 10.1073/pnas.0605134103 16880383 PMC1567706

[B28] Matrosovich M. Tuzikov A. Bovin N. Gambaryan A. Klimov A. Castrucci M. R. (2000). Early alterations of the receptor-binding properties of H1, H2, and H3 avian influenza virus hemagglutinins after their introduction into mammals. *J. Virol.* 74 8502–8512. 10.1128/JVI.74.18.8502-8512.2000 10954551 PMC116362

[B29] Matrosovich M. Zhou N. Kawaoka Y. Webster R. (1999). The surface glycoproteins of H5 influenza viruses isolated from humans, chickens, and wild aquatic birds have distinguishable properties. *J. Virol.* 73 1146–1155. 9882316 10.1128/jvi.73.2.1146-1155.1999 PMC103935

[B30] Mok C. K. Da Guan W. Liu X. Q. Lamers M. M. Li X. B. Wang M. (2015). Genetic characterization of highly pathogenic avian influenza A(H5N6) virus, Guangdong, China. *Emerg. Infect. Dis.* 21 2268–2271. 10.3201/eid2112.150809 26584075 PMC4672456

[B31] Naeve C. W. Hinshaw V. S. Webster R. G. (1984). Mutations in the hemagglutinin receptor-binding site can change the biological properties of an influenza virus. *J. Virol.* 51 567–569. 6748165 10.1128/jvi.51.2.567-569.1984 PMC254476

[B32] Ostrowsky B. Huang A. Terry W. Anton D. Brunagel B. Traynor L. (2012). Low pathogenic avian influenza A (H7N2) virus infection in immunocompromised adult, New York, USA, 2003. *Emerg. Infect. Dis.* 18 1128–1131. 10.3201/eid1807.111913 22710273 PMC3376803

[B33] Parrish C. R. Kawaoka Y. (2005). The origins of new pandemic viruses: the acquisition of new host ranges by canine parvovirus and influenza A viruses. *Annu. Rev. Microbiol.* 59 553–586. 10.1146/annurev.micro.59.030804.121059 16153179

[B34] Richard M. Herfst S. Tao H. Jacobs N. T. Lowen A. C. (2017). Influenza A virus reassortment is limited by anatomical compartmentalization following co-infection via distinct routes. *J. Virol.* 10.1128/JVI.02063-17 [Epub ahead of print]. 29212934 PMC5809721

[B35] Richard M. Schrauwen E. J. de Graaf M. Bestebroer T. M. Spronken M. I. van Boheemen S. (2013). Limited airborne transmission of H7N9 influenza A virus between ferrets. *Nature* 501 560–563. 10.1038/nature12476 23925116 PMC3819191

[B36] Rogers G. N. D’Souza B. L. (1989). Receptor binding properties of human and animal H1 influenza virus isolates. *Virology* 173 317–322. 10.1016/0042-6822(89)90249-3 2815586

[B37] Shi J. Deng G. Kong H. Gu C. Ma S. Yin X. (2017). H7N9 virulent mutants detected in chickens in China pose an increased threat to humans. *Cell Res.* 27 1409–1421. 10.1038/cr.2017.129 29151586 PMC5717404

[B38] Stevens J. Blixt O. Chen L. M. Donis R. O. Paulson J. C. Wilson I. A. (2008). Recent avian H5N1 viruses exhibit increased propensity for acquiring human receptor specificity. *J. Mol. Biol.* 381 1382–1394. 10.1016/j.jmb.2008.04.016 18672252 PMC2519951

[B39] Suzuki Y. Ito T. Suzuki T. Holland R. E. Chambers T. M. Kiso M. (2000). Sialic acid species as a determinant of the host range of influenza A viruses. *J. Virol.* 74 11825–11831. 10.1128/JVI.74.24.11825-11831.2000 11090182 PMC112465

[B40] Tong S. Zhu X. Li Y. Shi M. Zhang J. Bourgeois M. (2013). New world bats harbor diverse influenza A viruses. *PLoS Pathog* 9:e1003657. 10.1371/journal.ppat.1003657 24130481 PMC3794996

[B41] Watanabe T. Watanabe S. Maher E. A. Neumann G. Kawaoka Y. (2014). Pandemic potential of avian influenza A (H7N9) viruses. *Trends Microbiol.* 22 623–631. 10.1016/j.tim.2014.08.008 25264312 PMC4252989

[B42] Wu H. Lu R. Peng X. Chen B. Cheng L. Wu N. (2017). Molecular characterization of a novel reassortant H7N6 subtype avian influenza virus from poultry in Eastern China, in 2016. *Arch. Virol.* 162 1341–1347. 10.1007/s00705-017-3219-2 28105530

[B43] Yang W. Yin X. Guan L. Li M. Ma S. Shi J. (2018). A live attenuated vaccine prevents replication and transmission of H7N9 highly pathogenic influenza viruses in mammals. *Emerg. Microbes Infect.* 7:153. 10.1038/s41426-018-0154-6 30206210 PMC6133968

[B44] Yang Z. F. Mok C. K. Peiris J. S. Zhong N. S. (2015). Human infection with a novel avian influenza A(H5N6) virus. *N. Engl. J. Med.* 373 487–489. 10.1056/NEJMc1502983 26222578

[B45] Zhang C. Zhao Z. Guo Z. Zhang J. Li J. Yang Y. (2017). Amino acid substitutions associated with avian h5n6 influenza a virus adaptation to mice. *Front. Microbiol.* 8:1763. 10.3389/fmicb.2017.01763 28966609 PMC5605651

[B46] Zhang R. Chen T. Ou X. Liu R. Yang Y. Ye W. (2016). Clinical, epidemiological and virological characteristics of the first detected human case of avian influenza A(H5N6) virus. *Infect. Genet. Evol.* 40 236–242. 10.1016/j.meegid.2016.03.010 26973295 PMC4854533

[B47] Zhang Y. Zhang Q. Kong H. Jiang Y. Gao Y. Deng G. (2013). H5N1 hybrid viruses bearing 2009/H1N1 virus genes transmit in guinea pigs by respiratory droplet. *Science* 340 1459–1463. 10.1126/science.1229455 23641061

[B48] Zhao Z. Guo Z. Zhang C. Liu L. Chen L. Wang Z. (2017). Avian influenza H5N6 viruses exhibit differing pathogenicities and transmissibilities in mammals. *Sci. Rep.* 7:16280. 10.1038/s41598-017-16139-1 29176564 PMC5701206

